# Reward Responsiveness in the Adolescent Brain Cognitive Development (ABCD) Study: African Americans’ Diminished Returns of Parental Education

**DOI:** 10.3390/brainsci10060391

**Published:** 2020-06-19

**Authors:** Shervin Assari, Shanika Boyce, Golnoush Akhlaghipour, Mohsen Bazargan, Cleopatra H. Caldwell

**Affiliations:** 1 Department of Family Medicine, Charles R Drew University of Medicine and Science, Los Angeles, CA 90059, USA; mohsenbazargan@cdrewu.edu; 2 Department of Pediatrics, Charles R Drew University of Medicine and Science, Los Angeles, CA 90059, USA; ShanikaBoyce@cdrewu.edu; 3 Department of Neurology, University of California Los Angeles, Los Angeles, CA 90095, USA; Golnoush.akhlaghi@gmail.com; 4 Department of Family Medicine, University of California Los Angeles, Los Angeles, CA 90095, USA; 5 Center for Research on Ethnicity, Culture, and Health (CRECH), School of Public Health, University of Michigan, Ann Arbor, MI 48104, USA; cleoc@umich.edu; 6 Department of Health Behavior and Health Education, School of Public Health, University of Michigan, Ann Arbor, MI 48104, USA

**Keywords:** race, education, parenting, socioeconomic status, adolescents, reward, brain development, risk behaviors, cognition, brain, inhibitory control

## Abstract

(1) Background: Reward responsiveness (RR) is a risk factor for high-risk behaviors such as aggressive behaviors and early sexual initiation, which are all reported to be higher in African American and low socioeconomic status adolescents. At the same time, parental education is one of the main drivers of reward responsiveness among adolescents. It is still unknown if some of this racial and economic gap is attributed to weaker effects of parental education for African Americans, a pattern also called minorities’ diminished returns (MDRs). (2) Aim: We compared non-Hispanic White and African American adolescents for the effects of parent education on adolescents RR, a psychological and cognitive construct that is closely associated with high-risk behaviors such as the use of drugs, alcohol, and tobacco. (3) Methods: This was a cross-sectional analysis that included 7072 adolescents from the adolescent brain cognitive development (ABCD) study. The independent variable was parent education. The main outcome as adolescents’ RR measured by the behavioral inhibition system (BIS) and behavioral activation system (BAS) measure. (4) Results: In the overall sample, high parent education was associated with lower levels of RR. In the overall sample, we found a statistically significant interaction between race and parent education on adolescents’ RR. The observed statistical interaction term suggested that high parent education is associated with a weaker effect on RR for African American than non-Hispanic White adolescents. In race-stratified models, high parent education was only associated with lower RR for non-Hispanic White but not African American adolescents. (5) Conclusion: Parent education reduces RR for non-Hispanic White but not African American adolescents. To minimize the racial gap in brain development and risk-taking behaviors, we need to address societal barriers that diminish the returns of parent education and resources in African American families. We need public and social policies that target structural and societal barriers, such as the unequal distribution of opportunities and resources. To meet such an aim, we need to reduce the negative effects of social stratification, segregation, racism, and discrimination in the daily lives of African American parents and families. Through an approach like this, African American families and parents can effectively mobilize their resources and utilize their human capital to secure the best possible tangible outcomes for their adolescents.

## 1. Introduction

Reward responsiveness (RR) [1], a trait closely linked to impulsivity and risk taking [2], is a major driver of high-risk behaviors such as tobacco use [3,4,5,6,7,8], alcohol use [9,10,11,12], emotional eating [13], obesity [14], aggression [15], and sexual risk [16,17]. High RR is also associated with a wide range of psychiatric disorders such as depression, bipolar disorder, anxiety, and post-traumatic stress disorder (PTSD) [18]. Similar to the evidence that high-risk behaviors [19] and impulsivity [20,21] may be linked to race and socioeconomic status (SES), youth and adults with African American and low SES backgrounds may report higher RR than individuals from non-Hispanic White and high SES backgrounds [22].

Based on Gray’s reinforcement sensitivity theory (RST) [23], RR is one of the two neurobiological bases that guide human’s emotions, motivations, and behaviors. Rooted in the behavioral approach system (BAS) developed by the Carver and White [14], high RR reflects individuals’ high sensitivity to conditioned cues, which signal them about a higher-than-luck probability of reward. Individuals with a high score on the RR trait are more likely to act on any cues that may generate internal or external reward. In the recent version of the same theory [24], Gray and McNaughton have discussed BAS-based RR as well as approach-related behaviors and stimuli that contribute to human decisions, choices, and behaviors. Many investigators have found evidence linking the RR trait to a wide range of health and behavioral outcomes in clinical [22,25,26] as well as community [27] samples. RR is also highly relevant to adolescents’ behaviors and risk- taking [9,13,28].

Relative to their non-Hispanic White counterparts, African American adolescents are at an increased risk of high-risk behaviors. For example, African American adolescents are more likely than non-Hispanic White adolescents to be at risk of aggression [29], early sexual debut [30], and poor school performance [31]. As these early risk taking behaviors operate as a barrier against positive and desired health and economic outcomes later in life [32,33,34,35], it is essential to study environmental and psychological factors that explain high RR (and associated risky behaviors) of African American adolescents. Such knowledge may inform public and social policies as well as interventions that can be implemented during adolescence to eliminate later racial inequalities [32,33,34,35].

Given the close overlap between race and parental education in the US [36], researchers have shown immense interest in understanding the combined effects of race and parental education on adolescents’ inequalities [37,38,39]. As both racial minority status and low parental education reflect food and housing insecurity, economic adversities, stress, and financial difficulties [40,41,42,43], some of the effects of race may be in fact due to low parental education in African American families. Thus, low parental education may carry some of the effects of race on adolescents’ outcomes [36]. Recent data, however, show that the effects of race and parental education are more complex as they show both mediation and moderation effects on health inequalities [44,45,46,47]. While parental education is also a proxy of access to risk and protective factors [44,45,46,47], the protective effects of parental education seem to be weaker for African American than non-Hispanic White adolescents.

There are at least two complementary theories that provide an explanation for how race and parental education jointly impact adolescents’ outcomes. The first theory, dominant in the literature, and more commonly used as an explanation of the inequalities, attributes the observed racial gap in adolescents’ outcomes to the observed differences in parental education and other family SES indicators across racial groups [36,48,49,50]. In a statistical term, this theory conceptualizes parental education as the mediator (why) for racial differences in adolescents’ outcomes [51,52,53]. If this theory is followed, then the strategic goal for closing the racial gap in adolescents’ health would be to close the racial gap in family SES. Some example policy modalities in line with this strategy include income redistribution policies, minimum wage policies, or an earned income tax credit that help racial minorities to earn higher income and accumulate more wealth [54,55].

Minorities’ diminished returns (MDRs) [56,57], the second theory, however, argues that the effects of parental education and other family SES indicators tend to be weaker for racial minority groups such as African Americans, when compared to non-Hispanic Whites. This line of view is not against the traditional mediational model but provides an additional explanation for why, despite years of investment and the decline in the gap between races in terms of family SES, the racial and economic health gaps are still sustained and in some cases, widened [58,59,60,61,62]. The MDRs theory has been supported by a large number of papers showing that parental education [63], family income [64,65], and marital status [66] generate less health and well-being for African American than non-Hispanic White adolescents. This literature is repeatedly shown for emotional and behavioral outcomes [63,64,65,67,68]. For example, high family SES showed a smaller effect as a preventive factor on impulsivity [64], depression [67], anxiety [69], aggression [63], low grade point average (GPA) [63,70,71], and substance use [63] for African American than non-Hispanic White adolescents. Similarly, high SES African American youth are found to be at high risk of attention deficit hyperactive disorder (ADHD) [72] and obesity [73]. Given the existing MDRs, it would be too optimistic and unrealistic to expect racial inequities to disappear even if we could fully eliminate SES inequalities. In this view, SES indicators such as parental education are seen as both a remedy and also a source of inequalities across racial groups. While it is essential to eliminate the SES gap, we should complement our policy responses in a specific way that particularly empowers African American families to mobilize their resources and secure better health outcomes [56,57].

As described above, the MDR literature suggests that the educational attainment of oneself [74] and one’s parents [75,76,77] generate fewer tangible outcomes for racial minorities such as African Americans. This might be because African Americans and non-Hispanic Whites differ in getting chances and opportunities to mobilize their education and secure high paying jobs in the presence of high education [57,64,69,76,78,79]. As a result of these MDRs of parental education, compared to their non-Hispanic White counterparts, African American adolescents with highly educated parents show worse than expected outcomes that are disproportionate to their family SES [56,57,64,65,68]. Although these findings may be similar to what may be expected due regression to the mean, in a recent paper, we published and showed that MDRs are not due to such a superfluous association [80].

### Aims

To extend the science on what we already know about the role of RR as a mechanism for explaining MDRs for high-risk behaviors such as aggression, tobacco use, and impulsivity, in this study, we explored the combined effects of race and parent education on adolescents’ RR. Thus, we compared African American and non-Hispanic White adolescents for the effect of parent education, a strong family SES determinant of adolescents’ various behaviors, and RR. We expected a weaker effect of parent education on RR for African American than non-Hispanic White adolescents.

## 2. Methods

### 2.1. Design and Settings

We performed a secondary analysis of data from the adolescent brain cognitive development (ABCD) study [81,82,83,84,85], a landmark adolescents brain development study in the United States. Detailed information on the ABCD study is available elsewhere [81,86].

### 2.2. Participants and Sampling

Participants of the ABCD study were adolescents ages 9–10 years old. Adolescents in the ABCD study were recruited from multiple cities across states. Overall, there were 21 sites that recruited adolescents to the ABCD study. The recruitment of the ABCD sample was mainly done through school systems. A detailed description of the ABCD sampling is available here [87]. Four thousand one hundred eighty-eight participants entered our analysis. Eligibility for our analysis had valid data on race, parental education, marital status, RR, and being African American or non-Hispanic White. The analytical sample of this paper consisted of 7072 participants.

### 2.3. Study Variables

The study variables included race, demographic factors, parent education, parental marital status, and RR.

### 2.4. Outcome

Reward responsiveness (RR). In this study, RR was measured using the behavioral approach system (BAS) [1] developed by the Carver and White [14]. They define RR as a trait closely linked to impulsivity and risk taking [2], with a significant relevance to high-risk behaviors such as tobacco use [3,4,5,6,7,8], alcohol use [9,10,11,12], emotional eating [13], obesity [14], aggression [15], and sexual risk [16,17]. Based on Gray’s reinforcement sensitivity theory (RST) [23], a high score on the RR trait reflects individuals’ high sensitivity to conditioned cues, which signal the individual about a higher-than-luck probability of reward. We operationalized a BAS-based RR score, which was a continuous measure. Although BAS had other measures such as drive and fun seeking, we only used RR. This was because we built this study to examine effects on RR, not all BAS measures.

#### 2.4.1. Moderator: Race

Race was self-identified. Race was a categorical variable and coded 1 for African Americans and 0 for non-Hispanic Whites (reference category). All people of a Hispanic background were excluded. We used the one drop rule to handle people who identified as both White and African American. That means individuals would be considered African American if they identify as both African American and White.

#### 2.4.2. Independent Variable: Parent Education

Participants were asked, “What is the highest grade or level of school you have completed or the highest degree you have received?”. Responses were 0 = never attended/kindergarten only; 1 = 1st grade; 2 = 2nd grade; 3 = 3rd grade; 4 = 4th grade 4; 5 = 5th grade; 6 = 6th grade 6; 7 = 7th grade 7; 8 = 8th grade; 9 = 9th grade; 10 = 10th grade 10; 11 = 11th grade; 12 = 12th grade; 13 = high school graduate; 14 = GED or equivalent diploma; 15 = some college; 16 = associate degree: occupational; 17 = associate degree: academic program; 18 = Bachelor’s degree (ex. BA); 19 = Master’s degree (ex. MA); 20 = professional school degree (ex. MD); and 21 = Doctoral degree. This variable was coded in two distinct ways. First, it was coded as measured. This was an interval measure with a range between 1 and 21. Second, we adopted the Jaeger [88] coding approach with a range from 31 to 46. For both variables, a higher score indicated higher educational attainment.

#### 2.4.3. Confounders: Demographic Factors

Age, sex, parental marital status, and household size were the confounders. Parents reported the age of their adolescents. Age (years) was calculated as the difference between the date of birth to the date of the enrollment to the study. Sex was a dichotomous variable: males = 1, females = 0. Parental marital status was a dichotomous variable. This variable was self-reported by the parent who was interviewed. This variable was coded as married = 1 vs. other = 0. Household size, reported by the parent, was a continuous measure.

### 2.5. Data Analysis

We used the statistical package SPSS to perform our data analysis. Mean (standard deviation (SD)) and frequency (%) were described depending on the variable type. We also performed a Chi square and independent sample *t* test to test bivariate associations between race and study variables. For our multivariable modeling, we performed four linear regression models. Our first two models were performed in the overall sample. *Model 3* was performed without the interaction terms. *Model 4* added an interaction term between race and parental education attainment. Then we performed two additional models specifically to race (race-stratified models). *Model 3* was tested in non-Hispanic White and *Model 4* was tested in African American adolescents. Our models used age, sex, marital status, and household size as the covariates. We ran identical models using various coding of educational variable. Our first model used the census and our second variable used the Jaeger [88] code of educational attainment. Unstandardized regression coefficient (b), standardized regression coefficient, SE, 95% CI, *t* value, and *p* value were reported for each model. *p* value equal or less 0.05 were statistically significant.

### 2.6. Ethical Aspect

The ABCD study received an Institutional Review Board (IRB) approval from the University of California, San Diego (UCSD). Each adolescent provided assent. Each parent signed an informed consent [86]. As this analysis was performed on fully de-identified data, the study was found to be non-human subject research. Thus, our analysis was exempt from a full IRB review.

## 3. Results

### 3.1. Descriptives

As shown in Table 1, 7072, 8–11-year-old adolescents entered to this analysis. From this number, most were non-Hispanic Whites (*n* = 5099; 72.1%) and the rest were African Americans (*n* = 1973; 27.9%). Table 1 presents a summary of the descriptive statistics for the pooled sample.

### 3.2. Multivariate Analysis (Pooled Sample)

Table 2 shows the results of two linear regression models in the overall (pooled) sample. *Model 1* (the main effect model) showed a protective effect of high parent education against RR. *Model 2* (the interaction model) showed a statistically significant interaction between race and parent education on RR, which was suggestive of a weaker protective effect of high parent education on RR for African American adolescents relative to non-Hispanic White adolescents.

### 3.3. Multivariate Analysis (Race-Stratified Models)

Table 3 shows the results of two linear regression models in racial groups. *Model 3* (non-Hispanic Whites) showed protective effects of high parent education on RR of non-Hispanic White adolescents. *Model 4* (African Americans) did not show any protective effects of high parent education on RR for African American adolescents.

## 4. Discussion

In this study, while high parent education was associated with lower RR overall, this was only true for non-Hispanic White but not African American adolescents, as shown by the pooled sample as well as by race-stratified models. This suggests racial minority status limits the boosting effect of parent education on RR for American adolescents.

The observed diminished returns of parental education on RR is very similar to the previous publications on the MDRs of parental education and income on impulsivity [64], ADHD [72], and inhibitory control [89], social problems, emotional problems, behavioral problems [90], anxiety [69], depression [67], aggression [63], GPA [63,70,71], and substance use [63]. These are all examples of diminishing returns of parental education for African American youth when compared with non-Hispanic White youth [74,78,91,92].

MDRs are not specific to a specific resource or age group, outcome, or even marginalizing identities [56,57]. Education [74], income [64], employment [93], and marital status [69] show weaker effects on adolescents [64,65,68], adults [78], and older adults [94] who are African Americans [65], Hispanics [74,95,96,97], Asian Americans [98], Native Americans [99], lesbian, gay, bisexual, transgender, and queer (LGBTQ) [91], immigrants [100], or even marginalized non-Hispanic Whites [101].

Various sociological, economic, and behavioral mechanisms are involved in explaining the MDRs of parent education for African American families. African American parents and families experience high levels of economic, social, general, and race-related stress in their daily lives at all SES levels [102]. Racial groups do not have the same chance of upward social mobility in the US [103]. High SES African American families show an increase in exposure [104,105,106,107,108] and vulnerability [109] to discrimination, which may reduce the protective effects of SES on health. African American families with high SES are frequently surrounded by non-Hispanic Whites, which increases their exposure to discrimination [104,105]. Discrimination results in poor health across domains and limits the health gains that follow improving SES [107,109,110].

Residential segregation results in differences in African American and non-Hispanic White environmental and contextual exposures. Due to segregation, school options are different for high SES African American and Hispanic families. As a result, children of high SES African American families attend highly segregated schools with low resources [70,71,111]. That means there are differential effects of SES on education and schooling of non-Hispanic White and African American. While high SES non-Hispanic White adolescents attend schools in suburban areas with more funding and higher-quality teachers, African Americans go to schools that are of less quality [112].

While lower SES of African Americans is one type of disadvantage, MDRs reflect another class of disadvantage [56,57]. While the first one is about a lack of access to SES resources, MDRs are reflective of unequal outcomes despite access to SES. Thus, researchers and policymakers should not only address inequality in SES, but they should also address inequality in the returns of SES. African Americans are at a double disadvantage because they are affected by low SES and low return of the existing SES resources [56,113].

Multilevel economic, psychological, and societal mechanisms may be involved in explaining racial gaps in the returns of parental education [56,113]. MDRs may be due to racism across multiple societal institutions and social structures [56,113]. Racial prejudice interferes with the processes that are needed to gain benefits from available SES resources [114,115,116]. MDRs of educational attainment may be in part due to a history of childhood poverty [117]. The current study, however, did not explore societal and contextual processes that could explain such MDRs.

African American families are more likely to stay in poor neighborhoods despite high SES. Highly educated African Americans are more likely to stay poor than non-Hispanic Whites [77,118]. Similarly, African American families from high SES backgrounds are more likely to remain at risk of negative environmental exposures than non-Hispanic Whites with similar SES [104,105,107,119,120,121,122,123]. Similarly, high SES African American adolescents spend time with peers with higher risk and behavioral problems than non-Hispanic Whites with the same SES [63,98].

As this study showed, health disparities are not all due to SES differences, and some can be seen across all SES spectrum. This means, it is not race or SES but race and SES that shape health disparities [124,125,126]. The implication of these MDRs is that merely equalizing access to SES is not enough to tackle racial inequalities. Beyond attempts to eliminate or reduce SES gaps, there is a need to increase the degree to which SES can result in outcomes for African American families. To do so, policymakers should think about societal, environmental, and structural barriers that generate MDRs by reducing African Americas’ ability to leverage their resources. A real solution to MDRs-related disparities should be different from a solution to those who are caused by the SES-gaps. While the policy solution to health disparities due to SES gap is to increase African Americans’ access to SES resources, a true and sustainable remedy to the MDRs-related inequalities is to reduce structural barriers so African American families can efficiently and effectively translate their SES and human capital and secure tangible outcomes. This is not possible unless we equalize the daily living conditions of African Americans and non-Hispanic Whites.

While this study’s main association of interest was the effect of parental education on RR, we also found auxiliary results. We found results considering gender. In the pooled sample, boys showed a higher RR than girls. In Whites but not Blacks, males had higher RR. A higher reward responsiveness of males than females is known. This is also related to the higher impulsivity [127,128,129,130,131], reward dependence [16,132,133,134,135,136,137,138,139], and novelty seeking [140,141,142,143] of males than females. The result is a higher risk taking of males than females [127,144,145,146]. However, as mentioned before, this was not an exploratory study on correlates of RR. We were specifically interested in knowing if Black and White youth differ in the contribution of their parental educational attainment on their RR.

We also want to make it clear that MDRs are not due to Kelly’s Paradox [147] or regression toward the mean [148,149,150,151,152]. Under certain conditions, statistical artifacts, like regression to the mean or Kelly’s paradox, can produce similar results. However, in previous research [80], we showed that the MDRs were not attributable to statistical artifacts. While we do not verify that this is the case in the present study, we would argue, based on past results, that these MDRs represent the effects of the social environment. Kelly’s paradox [147], closely related to the regression to the mean [148,149,150,151,152], may occur when multiple groups with different starting points are compared. As described by Wainer and Brown (2007) [147], when a person from the poor-performing group exceeds the expectations, that person is expected to continue to overachieve, meaning he/she would perform even better. The opposite is also relevant to an underperformer in a high-performing group. In both cases, in reality, and opposite to the expectation, the individuals would regress toward their group means. That means, underperformers in a high-performing group and overperformers in a low-performing group are all more likely to have the average outcome rather than the expected outcome. In a recent paper, we have shown that MDRs are not due to regression toward the mean or Kelley’s paradox [147]. In fact, MDRs are not exclusively to the high-achiever or high-performance individuals, but any incremental increase in the resource generates less increase in the outcome for Blacks than Whites.

## 5. Limitations

This study, like any other studies, comes with a specific set of methodological limitations. As our data were cross-sectional in nature, we could not draw any causal inference between race, parent education, and adolescents’ RR. Similarly, we only tested the MDRs of parent education. Future research should test if MDRs go beyond parent education and hold for other SES indicators such as income, wealth, class, occupational prestige, and neighborhood SES indicators. Finally, this study only described the MDRs of parent education on RR and did not seek to understand the contextual factors that cause such MDRs.

In this study, RR [1] was measured using the behavioral approach, using BAS, which was developed by the Carver and White. We are unaware of any studies on the psychometric properties of this scale by race. So, we are not confident that the applied item measures identically the very same constructs in our race groups. So, there is a need to study if this measure is invariant across groups. As expected, we found a main effect of race on RR, suggesting that in line with the literature on associated traits such as impulsivity [64], RR is higher in Black than White youth. Future research should assess racial variation in measurement aspects of the BAS-based RR variable. Such effort would increase our confidence in comparing the results across groups and the observed means.

## 6. Conclusions

Relative to their non-Hispanic White counterparts, African American adolescents show lower parent education and higher RR. This adversity in African American youth is compounded by another profound and systemic disadvantage, weaker effects of parent education on adolescents RR. As a result of the latter disadvantage, African American adolescents show low RR across all parental education levels. That means some of the racial inequalities in RR remains across all educational levels. In other terms, racial inequalities in RR show a spill-over effect in middle-class people. As a result of high RR, African American adolescents engage in a high risk of behaviors across all levels of parental education. This is in contrast to the pattern for non-Hispanic White adolescents who show a social patterning of their RR. That is, for non-Hispanic White youth, RR is lowest when parental education is highest.

## Figures and Tables

**Table 1:** Data overall and by race (*n* = 7072).

Characteristics	All		Non-Hispanic Whites		African Americans	
	*n*	%	*n*	%	*n*	%
Race						
Non-Hispanic Whites	5099	72.1	5099	100.0	-	-
African Americans	1973	27.9	-	-	1973	100.0
Sex						
Male	3417	48.3	2432	47.7	985	49.9
Female	3655	51.7	2667	52.3	988	50.1
Marital Status *						
Other	2257	31.9	908	17.8	1349	68.4
Married	4815	68.1	4191	82.2	624	31.6
	Mean	SD	Mean	SD	Mean	SD
Age (Year)	9.47	0.51	9.47	0.50	9.47	0.52
Household Size	4.70	1.52	4.72	1.40	4.63	1.81
Parent education (Census Coding) *	16.92	2.40	17.55	2.00	15.30	2.57
Parent education (Jager Coding) *	42.06	2.20	42.61	1.87	40.58	2.23
Reward Responsiveness (RR) *	8.78	2.41	8.58	2.37	9.29	2.44

Note: SD = Standard deviation, * *p* < 0.05 for comparison of African American and non-Hispanic White adolescents.

**Table 2:** Summary of linear regressions overall (*n* = 7072).

	*Model 1* Main Effects						*Model 2* Interaction Effects					
Characteristics	b	SE	95% CI		T	*p*	B	SE	95% CI		*t*	*p*
Education (Jager Code)												
Race (African Americans)	0.61	0.09	0.43	0.79	6.53	<0.001	−2.67	1.56	−5.74	0.39	−1.71	0.087
Sex (Male)	0.31	0.07	0.17	0.45	4.44	<0.001	0.31	0.07	0.17	0.45	4.40	<0.001
Age	−0.02	0.07	−0.16	0.11	−0.31	0.760	−0.02	0.07	−0.16	0.11	−0.35	0.727
Married Household	−0.14	0.09	−0.33	0.04	−1.52	0.129	−0.15	0.09	−0.34	0.03	−1.64	0.101
Household Size	−0.03	0.02	−0.08	0.01	−1.37	0.171	−0.03	0.02	−0.08	0.01	−1.37	0.171
Parent Education	−0.07	0.02	−0.11	−0.04	−3.85	<0.001	−0.10	0.02	−0.14	− 0.05	−4.37	<0.001
Parent Education × African Americans	-	-	-	-	-	-	0.08	0.04	0.01	0.15	2.10	0.035
Constant	14.12	1.04	12.08	16.16	13.57	<0.001	15.28	1.18	12.97	17.59	12.99	<0.001
Education (Census Code)												
Race (African Americans)	0.53	0.08	0.38	0.68	6.98	<0.001	−0.34	0.45	−1.23	0.55	−0.75	0.454
Sex (Male)	0.33	0.06	0.21	0.44	5.69	<0.001	0.32	0.06	0.21	0.44	5.65	<0.001
Age	−0.05	0.06	−0.16	0.06	−0.88	0.381	−0.05	0.06	−0.16	0.06	−0.91	0.361
Married Household	−0.11	0.08	−0.26	0.04	−1.43	0.153	−0.12	0.08	−0.27	0.03	−1.51	0.131
Household Size	−0.03	0.02	−0.06	0.01	−1.33	0.185	−0.03	0.02	−0.07	0.01	−1.34	0.181
Parent Education	−0.05	0.01	−0.08	−0.03	−3.79	<0.001	−0.07	0.02	−0.11	−0.04	−4.22	<0.001
Parent Education × African Americans	-	-	-	-	-	-	0.05	0.03	0.00	0.11	1.95	0.050
Constant	10.03	0.59	8.86	11.19	16.90	<0.001	10.40	0.62	9.18	11.62	16.69	<0.001

Note: Unstandardized Regression Coefficient (b); Standard Error (SE); Confidence Interval (CI); Outcome: Reward Responsiveness (RR); *p* ≤ 0.050 considered significant.

**Table 3:** Summary of linear regressions between parental education and reward responsiveness (RR) by race (*n* = 7072).

	*Model 1* non-Hispanic Whites						*Model 2* African Americans					
Characteristics	B	SE	95% CI		T	*p*	b	SE	95% CI		*t*	*p*
Education (Jager Code)												
Sex (Male)	0.47	0.08	0.31	0.62	5.74	<0.001	−0.13	0.14	−0.40	0.14	−0.95	0.345
Age	−0.08	0.08	−0.23	0.08	−0.94	0.349	0.12	0.13	−0.14	0.39	0.91	0.365
Married Household	−0.23	0.11	−0.45	0.00	−2.00	0.0	−0.01	0.16	−0.33	0.31	−0.07	0.944
Household Size	−0.02	0.03	−0.08	0.04	−0.57	0.566	−0.06	0.04	−0.14	0.02	−1.48	0.138
Parent Education	−0.09	0.02	−0.14	−0.05	−4.10	<0.001	−0.03	0.03	−0.10	0.04	−0.91	0.364
Constant	15.42	1.25	12.97	17.87	12.35	<0.001	11.98	1.87	8.30	15.66	6.39	<0.001
Education (Census Code)												
Sex (Male)	0.47	0.07	0.34	0.60	7.18	<0.001	−0.09	0.11	− 0.32	0.13	−0.83	0.405
Age	−0.09	0.07	−0.22	0.04	−1.42	0.157	0.07	0.11	−0.15	0.29	0.61	0.539
Married Household	−0.17	0.09	−0.35	0.01	−1.80	0.072	−0.02	0.13	−0.28	0.25	−0.14	0.892
Household Size	−0.01	0.02	−0.06	0.04	−0.54	0.592	−0.05	0.03	−0.11	0.02	−1.47	0.141
Parent Education	−0.07	0.02	−0.10	−0.03	−3.97	<0.001	−0.03	0.02	−0.07	0.02	−1.05	0.296
Constant	10.62	0.70	9.24	11.99	15.14	<0.001	9.31	1.12	7.12	11.50	8.33	<0.001

Note: Unstandardized Regression Coefficient (b); Standard Error (SE); Confidence Interval (CI); Outcome: Reward Responsiveness (RR).
